# PLGA Nanoparticle-Mediated Sustained Release of Fisetin for Intra-Articular Therapy of Temporomandibular Joint Osteoarthritis

**DOI:** 10.3390/ijms27083618

**Published:** 2026-04-18

**Authors:** Ming Zhang, Jun-Ichiro Jo, Yoshiya Hashimoto, Yoshitomo Honda, Aki Nishiura

**Affiliations:** 1 Department of Orthodontics, Osaka Dental University, 8-1 Kuzuhahanazonocho, Hirakata 573-1121, Osaka, Japan; mingzhang1024@outlook.com (M.Z.); nishiura@cc.osaka-dent.ac.jp (A.N.); 2 Department of Biomaterials, Osaka Dental University, 8-1 Kuzuhahanazonocho, Hirakata 573-1121, Osaka, Japan; yoshiya@cc.osaka-dent.ac.jp; 3 Department of Oral Anatomy, Osaka Dental University, 8-1 Kuzuhahanazonocho, Hirakata 573-1121, Osaka, Japan; honda-y@cc.osaka-dent.ac.jp

**Keywords:** temporomandibular joint osteoarthritis, fisetin, nanoparticles, drug delivery systems, intra-articular injection, senescence

## Abstract

Temporomandibular joint osteoarthritis (TMJOA) is a degenerative maxillofacial disorder marked by progressive cartilage degradation and subchondral bone resorption, severely compromising patients’ quality of life. Intra-articular injection (IA), a standard route for conservative therapy, offers clinical advantages in safety and efficacy; however, outcomes remain limited due to short drug retention, poor tissue penetration, and variable agent efficacy, necessitating repeated administration. To overcome these limitations, fisetin-loaded poly (lactic-co-glycolic acid) nanoparticles (FST-PNP) were developed as a localized drug delivery system (DDS) for TMJOA treatment. Physicochemical analyses showed FST-PNP had uniform spherical morphology, excellent dispersibility, stability, high encapsulation efficiency, and substantial drug loading capacity. An in vitro study demonstrated more sustained and stable release from FST-PNP than free fisetin. The in vivo IA administration of FST-PNP preserved mandibular condylar osteochondral structures in TMJOA models. Notably, FST-PNP suppressed the expression of metalloproteinase-13 and a disintegrin and metalloproteinase with thrombospondin motifs-5 (ADAMTS5) as catabolic enzymes and downregulated p16 and p21 as senescence markers, indicating synergistic anti-inflammatory and anti-senescent effects. These findings highlight FST-PNP as a DDS integrating controlled-release with multifaceted therapeutic actions, providing a promising strategy for IA therapy of TMJOA.

## 1. Introduction

The temporomandibular joint (TMJ), the only bilaterally coordinated joint in humans, connects the skull and the mandible. It is essential for orofacial functions such as mastication, swallowing, and speech [1]. Temporomandibular joint osteoarthritis (TMJOA) is a prevalent chronic degenerative disorder characterized by synovitis, inflammation, extracellular matrix (ECM) degradation, aberrant angiogenesis, and dysregulated cellular activity [2,3,4,5]. Hallmark pathological features include condylar cartilage degeneration, osteochondral interface sclerosis, and subchondral bone resorption [6]. Clinically, TMJOA presents with pain, restricted mandibular motion, and joint noises, which considerably compromise patients’ quality of life [7,8].

Cellular senescence is closely associated with the onset and progression of osteoarthritis (OA) [9,10,11,12]. Inflammatory, oxidative, mechanical, and genomic stressors activate the DNA damage response (DDR), leading to cell cycle arrest and senescence through the p53–cyclin-dependent kinase inhibitor 1A (p21)–cyclin-dependent kinase 2 and cyclin-dependent kinase inhibitor 2A (p16)–cyclin-dependent kinase 4/6 [13]. Senescent cells accumulating in OA lesions secrete the senescence-associated secretory phenotype (SASP), comprising matrix metalloproteinase-13 (MMP13) and a disintegrin and metalloproteinase with thrombospondin motifs-5 (ADAMTS5), both central to TMJOA pathology. The SASP sustains a chronic inflammatory microenvironment and induces dysfunction in neighboring cells via paracrine signaling, creating a “senescence–inflammation–degeneration” cycle that drives OA progression [14,15]. Therefore, therapeutic strategies simultaneously targeting inflammation and senescence may help disrupt this vicious cycle in TMJOA.

Fisetin (3,7,3′,4′-tetrahydroxyflavone, FST), a natural flavonoid abundant in strawberries, grapes, and cucumbers [16], exhibits diverse bioactivities, including anti-inflammatory [17], anti-senescent [18,19,20,21], antioxidant [22], and antiangiogenic effects [23]. Recent studies indicate that FST activates silent information regulator 1 (SIRT1) to exert anti-inflammatory effects in OA [24] and inhibits chondrocyte senescence by targeting Sirtuin 6 (SIRT6) [25]. These findings highlight the therapeutic potential of FST as a dual anti-inflammatory and anti-senescent agent in TMJOA.

Currently, intra-articular injection (IA) is a widely used, safe, and effective approach for the conservative treatment of TMJOA [26]. However, IA-administered drugs are rapidly metabolized and cleared from the joint cavity, necessitating repeated dosing to maintain therapeutic levels. This limitation increases infection risk due to the cumulative microtrauma caused by repeated needle punctures [27], prolongs treatment duration, and yields variable efficacy with suboptimal overall outcome [28]. To overcome these limitations, drug delivery systems (DDSs) employing carriers with sustained and targeted release capabilities are pivotal for precise TMJ disease management [29]. Among these carriers, poly (lactic-co-glycolic acid) (PLGA), a biodegradable polymer with excellent biocompatibility, film-forming ability, and drug encapsulation capacity, has been extensively applied in biomedical contexts [30,31]. Following IA administration, PLGA nanoparticles enable sustained drug release governed by polymer degradation kinetics, thereby improving bioavailability, reducing dosing frequency, and enhancing therapeutic efficacy [32]. PLGA-based DDSs have shown promising outcomes in TMJ disorders, including mitigating synovitis, promoting tissue regeneration, and suppressing inflammation [33,34,35].

Preparation methods for FST-loaded PLGA nanoparticles (FST-PNP) are well established [36,37], markedly improving FST stability and bioavailability, which enhance its osteogenic and anticancer activities [38,39]. However, their therapeutic effects on TMJOA progression remain unexplored. This study reports the optimization and fabrication of FST-PNP, comprehensive characterization of their physicochemical properties and release profiles, evaluation of their anti-inflammatory and anti-senescent effects in an in vivo TMJOA model and assessment of their biosafety. This study aimed to establish the therapeutic potential and clinical applicability of FST-PNP for TMJOA.

## 2. Results and Discussion

### 2.1. FST-PNP Exhibits Structural Uniformity and Efficient Drug Encapsulation

**Figure 1:**
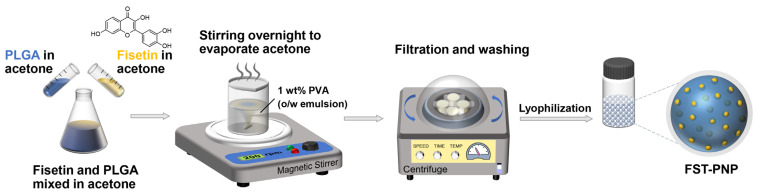
Schematic illustration of fisetin-loaded poly (lactic-co-glycolic acid) (PLGA) nanoparticle (FST-PNP) preparation using the single emulsion solvent evaporation (SESE) method. PLGA and fisetin in acetone were emulsified into 1 wt% polyvinyl alcohol (PVA) solution, stirred overnight for solvent evaporation, then collected by centrifugation, washed, and lyophilized.

The single emulsion solvent evaporation (SESE) method is widely employed to prepare polymeric nanoparticles (PNPs) for hydrophobic drug delivery, offering high drug-loading efficiency, controllable particle size, and broad applicability [40,41]. Emulsification conditions and material ratios have been optimized to improve PNP dispersibility, structural integrity, and stability, enhancing drug delivery efficiency [42]. In this study, FST-PNP were prepared by the SESE method using PLGA as the carrier (Figure 1).

**Figure 2:**
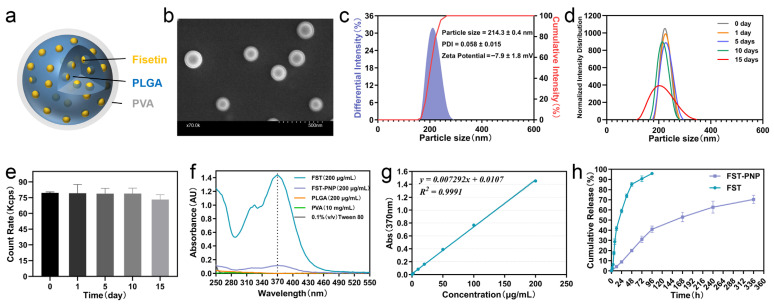
Characterization and release profile of FST-PNP. (**a**) Schematic illustration of FST-PNP structure; (**b**) scanning electron microscopy (SEM) image of FST-PNP. Scale bar: 500 nm; (**c**) particle size distribution and zeta potential of FST-PNP measured by dynamic light scattering (DLS) and electrophoretic light scattering (ELS); (**d**) hydrodynamic diameter stability of FST-PNP in PBS at 37 °C monitored over 15 days by DLS; (**e**) scattering intensity (count rate, kcps) of FST-PNP suspensions during 15-day incubation in PBS at 37 °C. Data are presented as mean ± SD (n = 3); (**f**) Ultraviolet-visible spectra of FST, FST-PNP, PLGA, PVA, and Tween 80; (**g**) standard calibration curve of free FST in PBS with 10% DMSO at 370 nm; (**h**) In vitro release profiles of free FST and FST-PNP in PBS containing 0.1% (*v*/*v*) Tween 80 at 4 °C over 14 days. Data are presented as mean ± SD (n = 3). Abbreviations: FST, fisetin; FST-PNP, fisetin-loaded poly (lactic-co-glycolic acid) nanoparticles; PLGA, poly (lactic-co-glycolic acid); PVA, polyvinyl alcohol; DMSO, dimethyl sulfoxide; SD, standard deviation; kcps, kilo-counts per second.

Scanning electron microscopy (SEM) revealed that the FST-PNPs exhibited a uniform spherical morphology with smooth surfaces and intact structures (Figure 2a,b). Dynamic light scattering (DLS) and electrophoretic light scattering (ELS) analyses showed a uniform particle size distribution (214.3 ± 0.4 nm) with a low polydispersity index (PDI = 0.058 ± 0.015) and a moderately negative surface charge (zeta potential = −7.9 ± 1.8 mV) (Figure 2c). When incubated for 15 days in phosphate-buffered saline (PBS, pH 7.4, 37 °C), particle size and scattering intensity remained stable (Figure 2d,e). Ultraviolet-visible spectrophotometry confirmed a specific absorption peak of FST at 370 nm (Figure 2f), with a highly linear absorbance–concentration relationship (Figure 2g). FST-PNP also displayed high encapsulation efficiency (EE = 100.58 ± 3.05%) and drug loading (DL = 7.04 ± 0.21%).

Compared with PLGA nanoparticles encapsulating fisetin, as reported by Dalle Carbonare et al. [38], (PDI = 0.11 ± 0.02, EE = 75.57 ± 4.21%); and Liu et al. [36], (PDI = 0.12 ± 0.01, EE = 79.3 ± 2.7%), the FST-PNP in this study demonstrated superior particle uniformity (lower PDI) and higher EE. These results indicate that FST-PNP possesses favorable physicochemical properties: uniform particle size and regular morphology facilitate predictable in vivo distribution, moderate negative surface charge and low PDI ensure colloidal stability, and high drug-loading capacity supports efficient in vivo delivery.

### 2.2. FST-PNP Demonstrates Controlled and Sustained Drug Release In Vitro

Multiple DDSs have been reported to achieve sustained FST release [43,44]. Consistent with these findings, our in vitro experiments (Figure 2h) demonstrated that FST-PNP markedly improved FST pharmacokinetics, exhibiting controlled and sustained release. In PBS at 4 °C, free FST reached 96% cumulative release by day 4 (96 h), whereas FST-PNP released only 41%, with sustained release extending beyond 14 days (336 h).

Due to the weak adsorption of some drug molecules on the nanoparticle surface, rapid desorption occurs upon contact with the release medium, resulting in a relatively faster release rate during the first four days, termed the “initial burst phase”. Subsequently, the encapsulated drug gradually diffuses outward, the release rate stabilizes, and the system enters the “sustained release phase” [45]. This process is regulated by polymer structure, hydrophilicity/hydrophobicity, and porosity [46]. Such a biphasic “burst-sustained” release profile allows FST-PNP to achieve sufficient early-stage drug concentration while maintaining long-term therapeutic effects, making it an ideal DDS for IA treatment of TMJOA.

### 2.3. FST-PNP Preserves Osteochondral Integrity in TMJOA

**Figure 3:**
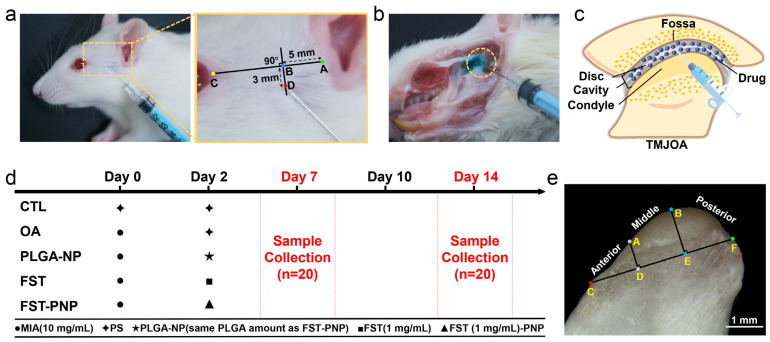
In vivo intra-articular injection procedure for treating monosodium iodoacetate (MIA)-induced temporomandibular joint osteoarthritis (TMJOA). (**a**) Anatomical location of the injection site on the body surface of a Sprague–Dawley rat; (**b**) gross anatomical image of the temporomandibular joint following intra-articular injection of Fast Green solution, verifying injection accuracy; (**c**) schematic diagram of drug distribution within the joint cavity; (**d**) treatment timeline with dosing and sample collection points; (**e**) division of the condyle into anterior, middle, and posterior slopes. Scale bar: 1 mm. Abbreviations: PS, physiological saline; CTL, control group; OA, osteoarthritis group; PLGA-NP, poly (lactic-co-glycolic acid) nanoparticles group; FST, fisetin group; FST-PNP, fisetin-loaded poly (lactic-co-glycolic acid) nanoparticles group.

The monosodium iodoacetate (MIA)-induced TMJOA model was successfully established, with Fast Green staining confirming accurate delivery of the solution into the joint cavity (Figure 3a,b). Diffusion of the injected solution is illustrated in Figure 3c. By using a structured experimental design and dividing the condyle into specific regions, a clear framework was provided for subsequent morphological and therapeutic evaluations of FST and FST-PNP treatments (Figure 3d,e).

**Figure 4:**
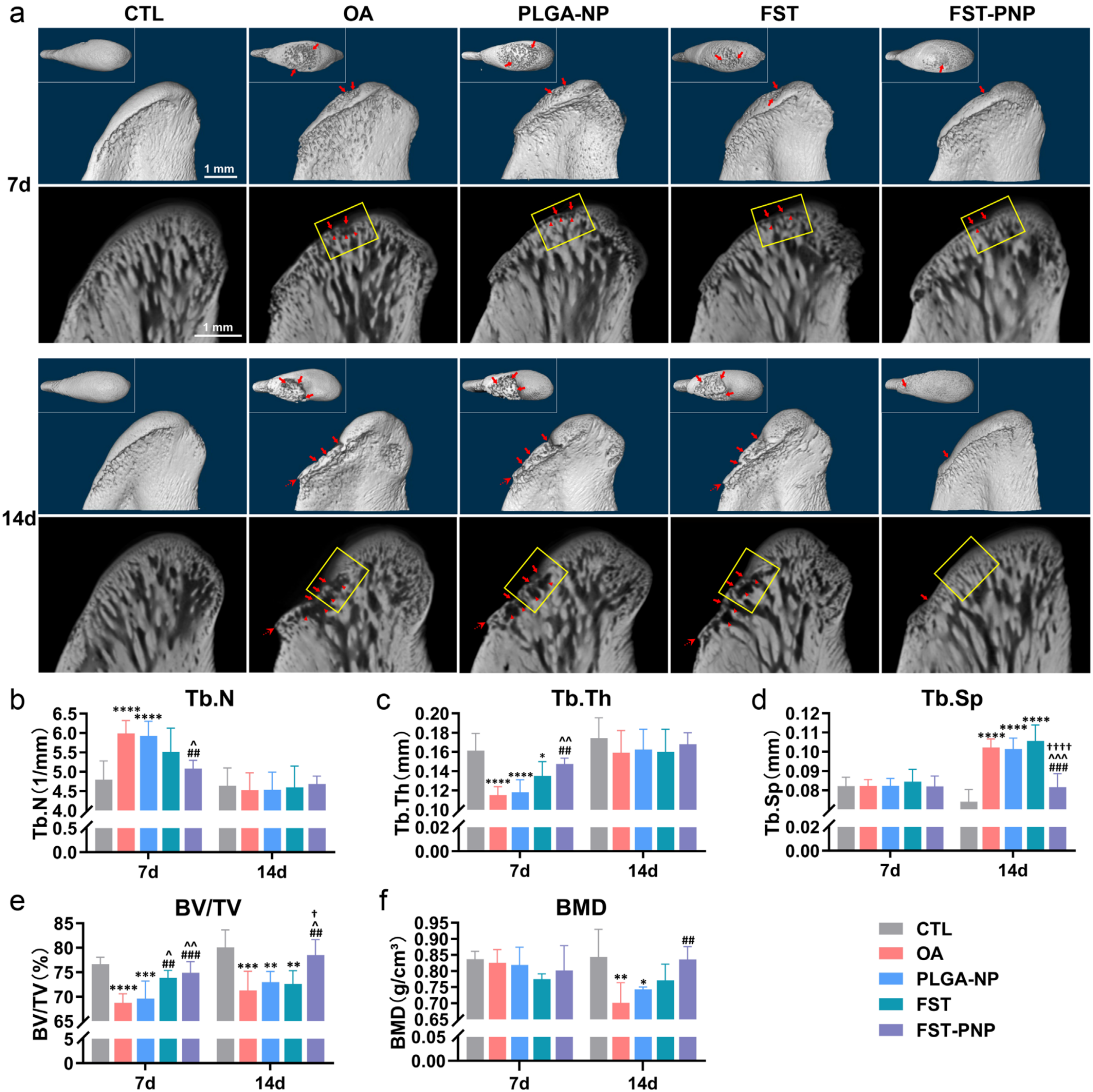
Micro-CT analysis of condylar tissue across groups to assess subchondral bone integrity and trabecular organization. TMJOA induction, treatment, and sample collection followed the schedule in Figure 3d. (**a**) Representative three-dimensional (3D) reconstructions and coronal micro-CT images of condyles. Yellow rectangles indicate the active osteochondral resorption area (AORA) on the middle slope; red arrows denote bone erosion; red triangles denote subchondral cysts; red dotted arrows indicate osteophytes. Scale bars: 1 mm (overview and inset); (**b**–**f**) quantitative micro-CT parameters: trabecular number (Tb.N), trabecular thickness (Tb.Th), trabecular separation (Tb.Sp), ratio of bone volume to total volume (BV/TV), and bone mineral density (BMD). Data are presented as mean ± SD (n = 6). * *p* < 0.05, ** *p* < 0.01, *** *p* < 0.001, **** *p* < 0.0001 vs. CTL; ## *p* < 0.01, ### *p* < 0.001 vs. OA; ^ *p* < 0.05, ^^ *p* < 0.01, ^^^ *p* < 0.001 vs. PLGA-NP; † *p* < 0.05, †††† *p* < 0.0001 vs. FST. Abbreviations: micro-CT, micro-computed tomography; TMJOA, Temporomandibular joint osteoarthritis; CTL, control group; OA, osteoarthritis group; PLGA-NP, poly (lactic-co-glycolic acid) nanoparticles group; FST, fisetin group; FST-PNP, fisetin-loaded poly (lactic-co-glycolic acid) nanoparticles group; SD, standard deviation.

To assess the effect of FST-PNP on the mandibular condylar subchondral bone, micro-computed tomography system (micro-CT) scanning and quantitative analysis were performed at days 7 and 14. Condylar lesions were mainly located on the anterior and middle slopes of the condyle, where mechanical stress is concentrated [47]. The active osteochondral resorption area (AORA) in the middle slope represents the transition from intact structure to erosive damage, reflecting dynamic lesion progression. On day 7, the OA group exhibited punctate bone erosion on the condylar surface, fragmented trabeculae, and small cystic changes. Concurrently, Tb.N increased, whereas Tb.Th and BV/TV decreased, indicating compensatory trabecular formation of suboptimal quality. By day 14, the OA group showed more pronounced erosive depressions, widened trabecular spacing, enlarged and fused cystic areas, and osteophyte formation. Tb.Sp was significantly increased, whereas BV/TV and BMD were markedly decreased. After IA treatment, the FST group showed partial alleviation of bone resorption on day 7, with increased BV/TV compared to the OA group, but there was little change on day 14. Conversely, the FST-PNP group demonstrated restoration of bone microstructural parameters to near-normal levels at days 7 and 14, with significantly reduced bone resorption. Notably, at day 14, compared with the FST group, the FST-PNP group showed significantly decreased Tb.Sp and increased BV/TV, indicating superior repair of subchondral bone microstructural damage (Figure 4a–f).

**Figure 5:**
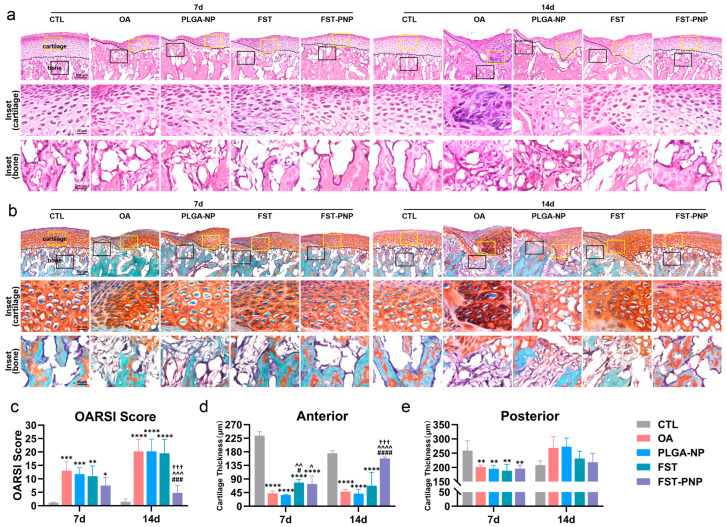
Histological evaluation of condylar cartilage degeneration and subchondral bone erosion across groups. TMJOA induction, treatment, and sample collection followed the schedule in Figure 2d. (**a**) Hematoxylin-eosin (H&E) staining and (**b**) safranin O-Fast green (SO-FCF) staining of the AORA. Yellow rectangles denote cartilage insets and black rectangles denote bone insets. Scale bars: 100 μm; insets, 25 μm; (**c**) Osteoarthritis Research Society International (OARSI) scoring of cartilage degeneration from SO-FCF-stained sections; (**d**,**e**) quantitative comparison of cartilage thickness at anterior and posterior AORA margins. Data are presented as mean ± SD (n = 4). * *p* < 0.05, ** *p* < 0.01, *** *p* < 0.001, **** *p* < 0.0001 vs. CTL; # *p* < 0.05, ### *p* < 0.001, #### *p* < 0.0001 vs. OA; ^ *p* < 0.05, ^^ *p* < 0.01, ^^^ *p* < 0.001, ^^^^ *p* < 0.0001 vs. PLGA-NP; ††† *p* < 0.001 vs. FST. Abbreviations: TMJOA, Temporomandibular joint osteoarthritis; AORA, active osteochondral resorption area; CTL, control group; OA, osteoarthritis group; PLGA-NP, poly (lactic-co-glycolic acid) nanoparticles group; FST, fisetin group; FST-PNP, fisetin-loaded poly (lactic-co-glycolic acid) nanoparticles group; SD, standard deviation.

Hematoxylin–eosin (H&E) and Safranin O–Fast Green (SO-FCF) staining further evaluated the therapeutic effect of FST-PNP on TMJOA-associated cartilage degeneration. The OA group displayed (Figure 5a,b) disorganized chondrocytes, localized defects, and uneven Safranin O staining, reflecting reduced proteoglycan content [48], with subchondral bone erosion accompanied by fibroblast-like cell infiltration. Conversely, the FST-PNP group exhibited markedly less cartilage degeneration. The Osteoarthritis Research Society International (OARSI) scores consistently revealed severe cartilage damage in the OA group. However, at day 14, the FST-PNP group exhibited substantially lower scores than the OA, PLGA-NP and FST groups (Figure 5c). Quantitative analysis of SO-FCF-stained images (Figure 5d,e) further showed that in the OA group, cartilage thickness decreased at the anterior margin at both day 7 and day 14, while the posterior margin exhibited thinning at day 7. This thickening likely reflects pathological remodeling of calcified cartilage, representing a dynamic balance between degeneration and compensatory repair during TMJOA progression [49,50]. However, the FST-PNP group exhibited sustained restoration of cartilage thickness at the anterior margin at day 14, with significantly greater recovery compared with the FST group. These results confirm the superior efficacy of FST-PNP in promoting cartilage repair.

MIA, an inhibitor of glyceraldehyde-3-phosphate dehydrogenase, disrupts chondrocyte glycolysis, triggering inflammatory responses and progressive joint degeneration, thereby inducing TMJOA [51]. Consistent with previous studies, the MIA-induced TMJOA model in this study showed lesions mainly localized in the condylar weight-bearing region, with severity worsening in a time-dependent manner, exhibiting typical imaging and histopathological characteristics of TMJOA, and demonstrating high reliability and reproducibility [52]. Previous animal studies have confirmed that FST promotes cartilage repair and improves subchondral bone structure [24,53]. In this study, the FST-PNP enhanced FST bioavailability in the TMJ cavity through sustained release, more effectively inhibiting TMJOA-associated osteochondral destruction and preserving osteochondral integrity.

### 2.4. FST-PNP Suppresses Inflammation and Reduces Cellular Senescence in TMJOA

During OA progression, inflammatory cytokines such as tumor necrosis factor-α (TNF-α) and interleukin-1β (IL-1β) considerably induce the expression of matrix-degrading enzymes MMP13 and ADAMTS5. These enzymes degrade the extracellular matrix, correlate with enhanced osteoclast activity, and disrupt subchondral bone homeostasis [54,55,56,57]. Notably, MMP13 and ADAMTS5 (essential components of the SASP) frequently act synergistically with senescence markers p16 and p21 in the cellular senescence process of TMJOA [58]. Therefore, by detecting these molecules, this study evaluated the anti-inflammatory and anti-senescent effects of FST-PNP.

**Figure 6:**
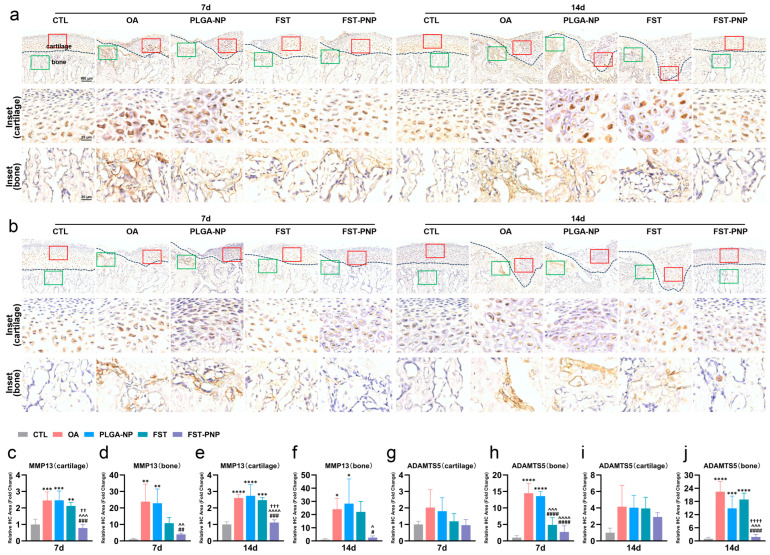
Immunohistochemical analysis of matrix degradation markers in condylar cartilage and subchondral bone. TMJOA induction, treatment, and sample collection followed the schedule in Figure 2d. (**a**) Immunohistochemical staining for MMP13 and (**b**) ADAMTS5 in the AORA. Red and green boxes denote cartilage and bone insets, respectively. Scale bars: 100 μm; insets, 25 μm; (**c**–**f**) quantification of MMP13-positive staining in cartilage and bone; (**g**–**j**) quantification of ADAMTS5-positive staining in cartilage and bone. Data are presented as mean ± SD (n = 4) and normalized to the CTL group (CTL = 1). * *p* < 0.05, ** *p* < 0.01, *** *p* < 0.001, **** *p* < 0.0001 vs. CTL; # *p* < 0.05, ## *p* < 0.01, ### *p* < 0.001, #### *p* < 0.0001 vs. OA; ^ *p* < 0.05, ^^ *p* < 0.01, ^^^ *p* < 0.001, ^^^^ *p* < 0.0001 vs. PLGA-NP; †† *p* < 0.01, ††† *p* < 0.001, †††† *p* < 0.0001 vs. FST. Abbreviations: TMJOA, temporomandibular joint osteoarthritis; AORA, active osteochondral resorption area; CTL, control group; OA, osteoarthritis group; PLGA-NP, poly (lactic-co-glycolic acid) nanoparticles group; FST, fisetin group; FST-PNP, fisetin-loaded poly (lactic-co-glycolic acid) nanoparticles group; SD, standard deviation; MMP13, matrix metalloproteinase-13; ADAMTS5, a disintegrin and metalloproteinase with thrombospondin motifs-5.

**Figure 7:**
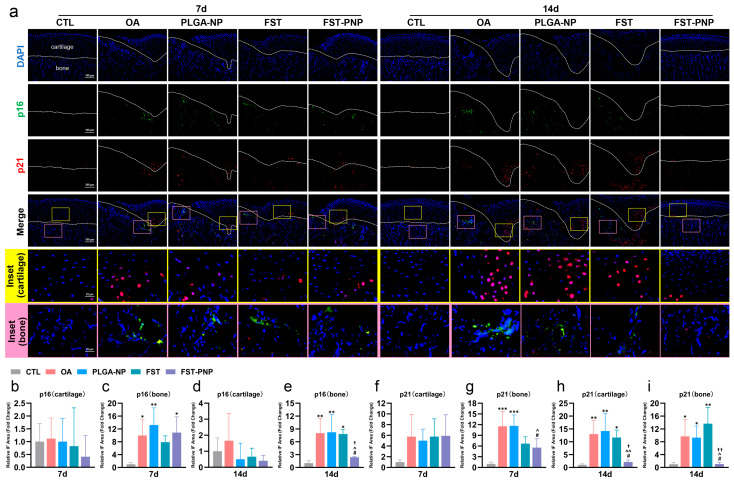
Immunofluorescence analysis of senescence markers in condylar cartilage and subchondral bone. TMJOA induction, treatment, and sample collection followed the schedule in Figure 2d. (**a**) Double immunofluorescence staining for p16 and p21 in the AORA. Yellow and pink boxes denote cartilage and bone insets, respectively. Scale bars: 100 μm; insets, 25 μm; (**b**–**e**) quantification of p16-positive staining in cartilage and bone; (**f**–**i**) quantification of p21-positive staining in cartilage and bone. Data are presented as mean ± SD (n = 4) and normalized to the CTL group (CTL = 1). * *p* < 0.05, ** *p* < 0.01, *** *p* < 0.001 vs. CTL; # *p* < 0.05 vs. OA; ^ *p* < 0.05, ^^ *p* < 0.01 vs. PLGA-NP; † *p* < 0.05, †† *p* < 0.01 vs. FST. Abbreviations: TMJOA, temporomandibular joint osteoarthritis; AORA, active osteochondral resorption area; CTL, control group; OA, osteoarthritis group; PLGA-NP, poly (lactic-co-glycolic acid) nanoparticles group; FST, fisetin group; FST-PNP, fisetin-loaded poly (lactic-co-glycolic acid) nanoparticles group; SD, standard deviation; p16, cyclin-dependent kinase inhibitor 2A; p21, cyclin-dependent kinase inhibitor 1A.

Immunohistochemical analysis revealed significantly elevated expression of MMP13 in cartilage and subchondral bone of the OA group (Figure 6a,c–f), as well as markedly increased ADAMTS5 expression in the subchondral bone (Figure 6b,g–j). Treatment with FST-PNP markedly reduced their levels, with a more pronounced inhibitory effect on MMP13 in cartilage and a stronger suppression of ADAMTS5 in subchondral bone at day 14 compared with the FST group. Immunofluorescence staining (Figure 7a–i) further showed that, compared with the control (CTL) group, both p16 and p21 were significantly upregulated in the subchondral bone at day 7 and day 14, while p21 was also markedly increased in cartilage at day 14. In addition, p21 was mainly localized to chondrocytes in the calcified cartilage layer. After FST-PNP treatment, p21 expression in the subchondral bone was significantly reduced at day 7. Compared with the FST group, both p16 and p21 expression were significantly suppressed at day 14. These findings indicate that FST-PNP exerts potent anti-inflammatory and anti-senescent effects in MIA-induced TMJOA, with greater efficacy than FST alone.

Consistent with these results, Wang et al. [25]. demonstrated that FST alleviates OA-related inflammation and cellular senescence by activating the SIRT6/Nrf2/HO-1 pathway, thereby downregulating MMP13, ADAMTS5, p16, and p21 in chondrocytes. Moreover, Nabizadeh et al. [59,60], due to sustained-release properties, developed chitosan nanoparticles and chitosan/hyaluronic acid hydrogels encapsulating FST, which showed superior inhibition of inflammatory cytokines and MMP13 compared with free FST. In the context of TMJOA, synovial inflammation is increasingly recognized as a critical factor in disease initiation and progression. Previous studies have suggested that the interplay between local joint injury and sustained inflammatory responses is required to drive degenerative changes within the joint [61]. This inflammatory microenvironment further contributes to cartilage degradation and cellular dysfunction, highlighting the importance of effective anti-inflammatory strategies. Similarly, this study demonstrates that in MIA-induced TMJOA, FST-PNP enhances the intra-articular bioavailability of FST through controlled release, thereby amplifying its dual anti-inflammatory and anti-senescent effects.

### 2.5. FST-PNP Exhibits Favorable In Vivo Biosafety

**Figure 8:**
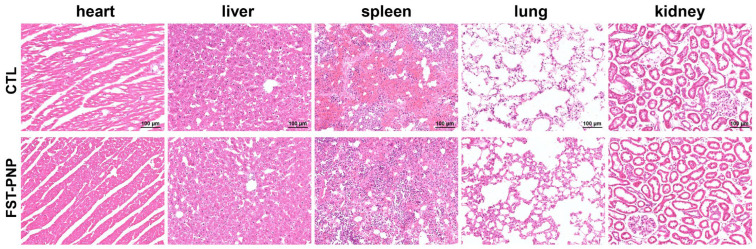
Biosafety evaluation of major organs by H&E staining after FST-PNP treatment. Scale bars: 100 μm. Abbreviations: CTL, control group; FST-PNP, fisetin-loaded poly (lactic-co-glycolic acid) nanoparticles group.

To evaluate the in vivo safety of FST-PNP, histological analyses were performed on major organs, including the heart, liver, spleen, lung, and kidney (Figure 8). H&E staining results showed no obvious pathological alterations in any of the examined groups.

In the heart, myocardial fibers were well organized without necrosis or inflammatory infiltration. In the liver, hepatocyte architecture remained intact without swelling, degeneration, or inflammatory response. The spleen showed normal architecture with clear white and red pulp and no lymphoid hyperplasia. The lung exhibited preserved alveolar structure without septal thickening or inflammation. The kidney showed normal glomeruli and intact renal tubules without degeneration or necrosis. Overall, no significant histopathological damage was observed in major organs following FST-PNP treatment.

From the perspective of biomaterials and drug delivery systems, systemic toxicity is a critical parameter for evaluating clinical translational potential. These results demonstrate that FST-PNP exhibits favorable in vivo biocompatibility and safety, providing important evidence supporting its application for TMJOA therapy.

Although this study systematically evaluated the protective effects of FST-PNP on condylar osteochondral structures, and its anti-inflammatory and anti-senescent actions in MIA-induced TMJOA, the precise mechanisms remain unclear, particularly how FST regulates cellular interactions and homeostasis within the condylar osteochondral microenvironment. The long-term metabolism, distribution, and safety of FST-PNP in the TMJ cavity warrant further investigation, which is crucial for clinical translation. Future research should integrate in vitro mechanistic study, pathway analysis, and multi-omics approaches to elucidate the key signaling pathways of FST in condylar osteochondral tissue and apply fluorescent or radioactive labeling to track the dynamic behavior of FST-PNP in the TMJ cavity to optimize dosing. Furthermore, combining FST-PNP with other therapeutic modalities may provide more effective precision treatments for TMJOA.

## 3. Materials and Methods

### 3.1. Preparation of FST-PNP

FST-PNP was prepared using the SESE method (Figure 1). Briefly, 100 mg of PLGA (molecular weight: 15 kDa, lactide/glycolide ratio: 50:50, FUJIFILM Wako Pure Chemical Corporation, Osaka, Japan) and 2.8 mg FST (Tokyo Chemical Industry Co., Ltd., Tokyo, Japan) were separately dissolved in 1 mL of acetone (Nacalai Tesque Inc., Kyoto, Japan). A mixed organic phase was prepared by combining 0.2 mL PLGA solution, 0.5 mL FST solution, and 0.3 mL acetone. Under 25 °C and 200 rpm, this phase was added dropwise into 10 mL of 1 wt% polyvinyl alcohol (PVA, JP-10, Vam & Poval Co., Ltd., Osaka, Japan) aqueous solution, followed by overnight stirring to evaporate acetone, yielding an FST-PNP suspension. The suspension was centrifuged (4 °C, 15,000 rpm, 30 min), the supernatant discarded, and the pellet resuspended in ultrapure water. Washing was repeated three times, and the final product was freeze-dried and stored at −20 °C.

### 3.2. Characterizations of FST-PNP

#### 3.2.1. Morphological Analysis

Surface morphology was assessed using SEM (S-4800, Hitachi High-Tech Corporation, Tokyo, Japan). Carbon double-sided tape (Nisshin-EM Co., Ltd., Tokyo, Japan) was affixed to a specimen mount (S-HM, Nisshin-EM Co., Ltd.), followed by an aluminum foil sheet. Subsequently, 10 μL of FST-PNP suspension (0.2 mg/mL in ultrapure water) was dried onto the foil. Samples were sputter-coated with a thin metal layer (HPC-20, Vacuum Device Inc., Ibaraki, Japan), mounted in the SEM chamber, and imaged at 5.0 kV accelerating voltage and 10 μA current.

#### 3.2.2. Particle Size, Polydispersity Index, and Zeta Potential

Size and PDI of FST-PNP (0.2 mg/mL in ultrapure water) were measured at 25 °C using DLS on a zeta potential and particle size analyzer (ELSZ-2000, Otsuka Electronics Co., Ltd., Osaka, Japan). Zeta potential was determined by ELS.

#### 3.2.3. Stability Evaluation

To assess stability under physiological conditions, FST-PNP samples (0.2 mg/mL) were dispersed in PBS (pH 7.4, FUJIFILM Wako Pure Chemical Corporation) and incubated at 37 °C for 0, 1, 5, 10, and 15 days. Particle size and scattering intensity were measured at each time point to monitor the stability.

#### 3.2.4. Encapsulation Efficiency and Drug Loading

To ensure accurate FST quantification and evaluate potential interference from each component, the absorbance at 370 nm of FST, FST-PNP, PLGA, PVA, and Tween 80 was measured using an ultraviolet-visible spectrophotometer (ND-1000, Thermo Fisher Scientific Inc., Waltham, MA, USA). FST standard solutions (0.4–200 μg/mL) were prepared in PBS containing 10% dimethyl sulfoxide (DMSO, FUJIFILM Wako Pure Chemical Corporation), and their absorbance at 370 nm was recorded. A standard calibration curve was constructed, and linear regression provided the regression equation and correlation coefficient (R^2^). FST-PNP was dissolved in PBS with 10% DMSO, and its absorbance at 370 nm was measured. Encapsulation efficiency (EE) and drug loading (DL) were calculated using the standard curve according to the following equations. Here, *FST_fed_* denotes the initial amount of fisetin introduced during nanoparticle formulation, and *FST_loaded_* represents the actual amount encapsulated within the nanoparticles.

(1) $$EE\left(\%\right)=\frac{{FST}_{loaded}}{{FST}_{fed}}\times 100,$$

(2) $$DL\left(\%\right)=\frac{weight\;of\;{FST}_{loaded}}{weight\;of\;PLGA}\times 100.$$

### 3.3. In Vitro Release Profile of FST-PNP

The in vitro release of FST-PNP was evaluated using the dialysis method. Free FST or FST-PNP containing 1 mg of FST was suspended in 500 μL PBS and placed in a dialysis bag (MWCO 14,000 Da, Sigma-Aldrich Inc., St. Louis, MO, USA). The sealed bags were immersed in 100 mL of PBS containing 0.1% (*v*/*v*) Tween 80 (Sigma-Aldrich Inc.) and stirred at 200 rpm at 4 °C. At predetermined time points, 900 μL of release medium was collected and mixed with 100 μL DMSO, and absorbance at 370 nm was measured. An equal volume of fresh medium was added to maintain constant volume and sink conditions. FST concentrations were calculated from the standard curve, and cumulative release was plotted over time.

### 3.4. Induction and Treatment of the Rat TMJOA Model

All animal procedures were approved by the Animal Care and Use Committee of Osaka Dental University (Approval No. 25-01001) and conducted in strict accordance with the American Veterinary Medical Association guidelines, following the 3R principles. Eight-week-old male Sprague–Dawley rats (Shimizu Laboratory Supplies Co., Ltd., Kyoto, Japan) were initially anesthetized via inhalation of isoflurane (5% induction, 1 L/min O_2_). Once the righting reflex was lost, deep anesthesia was maintained by intraperitoneal injection of a composite anesthetic (0.5 mL per rat; containing etorphine 2.5 mL, medetomidine 0.75 mL, and midazolam 2 mL, brought to a total volume of 10 mL with sterile water), with anesthesia depth confirmed by the absence of corneal and paw withdrawal reflexes. TMJOA was induced by injection of 50 μL MIA (10 mg/mL in saline, Sigma-Aldrich Inc.) into each TMJ. The injection method [62] was as follows (Figure 3a): a baseline was drawn between the preauricular point (A) and the postorbital angle point (C). Point B was marked 5 mm anterior to point A, and the injection site (D) was located 3 mm vertically below point B. A needle was inserted from point D at a 45° angle in the upward, forward, and medial directions, passing beneath the zygomatic arch until contacting the articular fossa of the temporal bone, followed by slow injection. Injection accuracy was verified by Fast Green dye (ScienCell Research Laboratories Inc., Carlsbad, CA, USA) using the same method, with uniform dye distribution confirmed after dissection (Figure 3b). Rats were randomly assigned to five groups (n = 8/group): CTL, TMJOA, PLGA-NP, FST, and FST-PNP (Figure 3d). All groups received bilateral IAs (50 μL/side). TMJOA was induced on day 0, and treatment was administered on day 2. On days 7 and 14, rats were euthanized, first by deep anesthesia with isoflurane and then by cervical dislocation, after which condylar tissues were immediately harvested (Figure 3d). Death was confirmed by the absence of heartbeat and corneal reflexes. Samples were fixed in 4% paraformaldehyde phosphate buffer (Nacalai Tesque Co., Ltd.) at 4 °C. Condylar morphology was observed with a stereomicroscope (SZX12, Olympus Corporation, Tokyo, Japan). The condylar slope was divided into anterior, middle, and posterior regions based on anatomical landmarks for subsequent analyses (Figure 3e).

### 3.5. Micro-Computed Tomography Analysis

Six samples per group were scanned with a micro-CT (SKYSCAN 1275, Bruker Co., Billerica, MA, USA) at 40 kV and 50 μA without a filter. Samples were mounted on a 25 mm diameter holder. Images were reconstructed with NRecon software (version 1.7.4.2, Bruker Co.) and reoriented in coronal sections using DataViewer software (version 1.5.6.2, Bruker Co.). Quantitative analysis was performed with CTAn software (version 1.17.4.2, Bruker Co.) using a uniform grayscale threshold (low: 60, high: 140). The following trabecular bone parameters were measured: trabecular number (Tb.N), trabecular thickness (Tb.Th), trabecular separation (Tb.Sp), bone volume fraction (bone volume/total volume; BV/TV), and bone mineral density (BMD). Three-dimensional visualization was generated with CTvox software (version 3.3.0r1403, Bruker Co.).

### 3.6. Histological Evaluation

Samples were decalcified in ethylenediamine tetraacetic acid (EDTA) solution (FUJIFILM Wako Pure Chemical Corporation) and dehydrated through a sucrose gradient (Nacalai Tesque Co., Ltd.). Frozen sections were prepared using the Kawamoto method [63]. H&E and SO-FCF (ScienCell Research Laboratories Inc., Carlsbad, CA, USA) stainings were performed. The AORA in the middle condylar slope was imaged under a microscope (BZ-X800, Keyence Corporation, Osaka, Japan). Three blinded observers graded cartilage degeneration using the OARSI scoring system [64]. Based on SO–FCF images, cartilage thickness at the anterior and posterior AORA margins was quantified using ImageJ software (version 1.53, National Institutes of Health, Bethesda, MD, USA).

### 3.7. Immunohistochemical Evaluation

Sections underwent antigen retrieval at 70 °C in retrieval solution (Nacalai Tesque Co., Ltd.), permeabilization with 0.3% Triton X-100 (Nacalai Tesque Co., Ltd.) for 10 min, and peroxidase blocking with 3% hydrogen peroxide (MUTO PURE CHEMICALS Co., Ltd., Tokyo, Japan) for 10 min. After 30 min blocking (Nacalai Tesque Co., Ltd.), sections were incubated overnight at 4 °C with primary antibodies against MMP13 (1:200; bs-10581R, Bioss Inc., Woburn, MA, USA) or ADAMTS5 (1:100; DF13268, Affinity Biosciences Inc., Cincinnati, OH, USA). Following washes, horseradish peroxidase (HRP)-conjugated secondary antibody (1:200; S0001, Affinity Biosciences Inc., Cincinnati, OH, USA) was applied for 40 min at room temperature. Immunoreactivity was visualized with a 3,3′-diaminobenzidine (DAB) substrate kit (Nacalai Tesque Co., Ltd.), counterstained with hematoxylin, and mounted. Images of AORA were acquired, and positive staining areas were quantified using ImageJ.

### 3.8. Immunofluorescence Evaluation

As described above, sections underwent antigen retrieval, permeabilization, and blocking. Primary antibodies against p16 (1:100; bs-23797R-A488, Bioss Inc., Woburn, MA, USA) or p21 (1:100; bs-10129R-BF555, Bioss Inc., Woburn, MA, USA) were applied overnight at 4 °C. After washing, sections were mounted with DAPI Fluoromount (Nacalai Tesque Co., Ltd.). Images of the AORA were captured, and the percentage of positive staining was quantified using ImageJ.

### 3.9. In Vivo Biosafety Evaluation

Rats were assigned to CTL and FST-PNP groups. The CTL group received IAs of saline, whereas the FST-PNP group received FST-PNP, using the same dosage and administration protocol as in the TMJOA therapeutic experiments. At 4 weeks after administration, rats were euthanized, and major organs (heart, liver, spleen, lung, and kidney) were harvested, fixed in 4% paraformaldehyde, and dehydrated through a sucrose gradient. Frozen sections were prepared and stained with H&E. Histopathological evaluation was performed to assess in vivo biosafety.

### 3.10. Statistical Analysis

Data are presented as mean ± standard deviation (SD). One-way analysis of variance (one-way ANOVA) was used for statistical comparisons between groups, followed by Tukey’s post hoc test for multiple comparisons. Analyses were conducted using GraphPad Prism software (version 10.1.2; GraphPad Software Inc., San Diego, CA, USA). Statistical significance was set at *p* < 0.05.

## 4. Conclusions

In this study, FST-PNPs with favorable physicochemical properties were prepared and comprehensively evaluated for therapeutic efficacy in MIA-induced TMJOA. FST-PNP achieved sustained FST release, promoted repair of condylar osteochondral structures, and suppressed inflammation and cellular senescence. In addition, histological evaluation of major organs revealed no obvious pathological alterations after FST-PNP treatment, indicating favorable in vivo biosafety. Compared with FST, FST-PNP demonstrated superior bioavailability and therapeutic outcomes, highlighting its potential as an intra-articular DDS. Integrating mechanistic studies with delivery system optimization may accelerate the clinical translation of FST-PNP for the precision treatment of TMJOA.

## Data Availability

The data that support the findings of this study are available from the corresponding author, J.-I.J., upon reasonable request because the dataset includes detailed experimental parameters that are closely related to ongoing and future research.
